# Lactate Induces the Expressions of MCT1 and HCAR1 to Promote Tumor Growth and Progression in Glioblastoma

**DOI:** 10.3389/fonc.2022.871798

**Published:** 2022-04-28

**Authors:** Lucia Longhitano, Nunzio Vicario, Daniele Tibullo, Cesarina Giallongo, Giuseppe Broggi, Rosario Caltabiano, Giuseppe Maria Vincenzo Barbagallo, Roberto Altieri, Marta Baghini, Michelino Di Rosa, Rosalba Parenti, Antonio Giordano, Maria Caterina Mione, Giovanni Li Volti

**Affiliations:** ^1^Department of Biomedical and Biotechnological Sciences, University of Catania, Catania, Italy; ^2^Department of Medical and Surgical Sciences and Advanced Technologies “G.F. Ingrassia”, Catania, Italy; ^3^Department of Medical and Surgical Sciences and Advanced Technologies “G.F. Ingrassia” Neurological Surgery, Policlinico “G. Rodolico-San Marco” University Hospital, University of Catania, Catania, Italy; ^4^Interdisciplinary Research Center on Brain Tumors Diagnosis and Treatment, University of Catania, Catania, Italy; ^5^Department of Cellular, Computational and Integrative Biology (CIBIO), University of Trento, Trento, Italy; ^6^Sbarro Institute for Cancer Research and Molecular Medicine and Center of Biotechnology, College of Science and Technology, Temple University, Philadelphia, PA, United States

**Keywords:** lactate, glioblastoma, MCT1 (SLC16A1), HCAR1, metabolism

## Abstract

The tumor microenvironment (TME) plays a pivotal role in establishing malignancy, and it is associated with high glycolytic metabolism and lactate release through monocarboxylate transporters (MCTs). Several lines of evidence suggest that lactate also serves as a signaling molecule through its receptor hydroxycarboxylic acid receptor 1 (HCAR1/GPR81), thus functioning as a paracrine and autocrine signaling molecule. The aim of the present study was to investigate the role of lactate in glioblastoma (GBM) progression and metabolic reprogramming in an *in vitro* and *in vivo* model. The cell proliferation, migration, and clonogenicity were tested *in vitro* in three different human GBM cell lines. The expressions of *MCT1*, *MCT4*, and *HCAR1* were evaluated both *in vitro* and in a zebrafish GBM model. The results were further validated in patient-derived GBM biopsies. Our results showed that lactate significantly increased the cell proliferation, migration, and colony formation capacity of GBM cells, both *in vitro* and *in vivo*. We also showed that lactate increased the expressions of *MCT1* and *HCAR1*. Moreover, lactate modulated the epithelial–mesenchymal transition protein markers E-cadherin and β-catenin. Interestingly, lactate induced mitochondrial mass and the *OXPHOS* gene, suggesting improved mitochondrial fitness. Similar effects were observed after treatment with 3,5-dihydroxybenzoic acid, a known agonist of *HCAR1*. Consistently, the GBM zebrafish model exhibited an altered metabolism and increased expressions of *MCT1* and *HCAR1*, leading to high levels of extracellular lactate and, thus, supporting tumor cell proliferation. Our data from human GBM biopsies also showed that, in high proliferative GBM biopsies, Ki67-positive cells expressed significantly higher levels of *MCT1* compared to low proliferative GBM cells. In conclusion, our data suggest that lactate and its transporter and receptor play a major role in GBM proliferation and migration, thus representing a potential target for new therapeutic strategies to counteract tumor progression and recurrence.

## Introduction

Glioblastoma (GBM) represents the most common primary brain tumor in the adult population and is classified by the WHO as grade IV glioma. Current therapeutic approaches for newly diagnosed GBM include surgical resection, radiotherapy, and chemotherapy (i.e., temozolomide) (1). However, despite aggressive therapeutic regimens, these tumors still have a dismal prognosis, with median overall survival of 12–15 months. Histologically, GBM is a highly cellular glioma composed of glial cells with significant pleomorphism and nuclear atypia (2). Such cellular features are coupled with microvascular proliferation and palisading necrosis characterized by regular areas of necrosis and dense accumulation of GBM cells (2). The characteristics of GBM are related to cell proliferation, usually assessed by evaluating Ki67-expressing cells classified into high proliferative index (HPI; Ki67-positive cells >30%) and low proliferative index (LPI; Ki67-positive cells <30%). Furthermore, the proliferation, migration, and invasiveness of GBM cells are closely related to the availability of blood-derived nutrients and oxygen. Indeed, two niches have been described in GBM in relation to the availability of oxygen: the so-called perivascular niche, in which GBM cells receive glucose and oxygen from the bloodstream and oxidative phosphorylation in these cells determines a highly efficient metabolism, and the GBM hypoxic niche at the tumor core, in which low oxygen levels shape the metabolism toward a glycolytic state, inducing lactate accumulation (3). Indeed, such tumors have a rapid rate of glucose consumption and convert large amounts of glucose into lactic acid, even in the presence of oxygen (4). This metabolic phenotype, known as the Warburg effect, contrasts sharply with that observed in normal tissues, in which glycolysis occurs mainly in hypoxic conditions (5).

To maintain an enhanced glycolytic flow, GBMs require the rapid outflow of lactic acid into the tumor microenvironment (TME), facilitated by a series of plasma membrane transporters called monocarboxylate transporters (MCTs) (6); among these, only four (MCT1–4) are known to play a role in lactic acid transport in mammalian tissues, including cancers (7), and *MCT1* and *MCT4* have been implicated in multiple aspects of GBM progression, including angiogenesis, cell proliferation, and immunity modulation (8). Glycolytic cancer cells are known to upregulate lactate export by increasing the expression of *MCT4* to better adapt to lactate accumulation. In contrast, tumor cells of oxidative tumors have been reported to upregulate the expression of *MCT1* to mediate the uptake of lactate from the extracellular environment in order to fuel metabolism (9). A recent report has suggested that this dynamic arrangement may create a metabolic symbiosis between the two GBM subpopulations, maintaining a favorable environment for both subtypes (8, 10).

Besides having a role as an end-product metabolite of glycolysis and being utilized by cellular metabolic programs to produce energy, lactate also acts as a signaling molecule through its receptor hydroxycarboxylic acid receptor 1 (*HCAR1*; also known as G-protein-coupled receptor GPR81) (11). Therefore, extracellular lactate is not a simple bystander causing milieu acidification; it also serves as a paracrine and autocrine signaling molecule in the TME (12). An elevated expression of *HCAR1* was found in carcinomas of the breast, pancreas, and cervix, despite negligible expression in the corresponding benign epithelium (12, 13). Several groups have identified autocrine roles for *HCAR1* in the TME, where lactate produced by tumor cells activates *HCAR1* and confers cancer-promoting phenotypes (14), including the upregulation of the transporters *MCT1* and *MCT4* and the secretion of factors that promote angiogenesis and tumor progression (15). The aim of the present study was to assess the role of lactate metabolism in cancer growth and progression in several GBM cell lines in pathological specimens and in an *in vivo* model.

## Materials and Methods

### GBM Cell Lines

Human GBM cell lines (U87-MG, A172, and U251) were purchased from ATCC Company (Milan, Italy). Cells were suspended in Dulbecco’s modified Eagle’s medium (DMEM) (cat. no. 11965092) containing 10% fetal bovine serum (FBS) (cat. no. 10082147), 100 U/ml penicillin, and 100 U/ml streptomycin (cat. no. 15070063; all from Gibco, Waltham, MA, USA). At 80% confluency, cells were passaged using trypsin–EDTA solution (0.05% trypsin and 0.02% EDTA; cat. no. 25300054, Gibco, Waltham, MA, USA).

Sodium lactate and 3,5-dihydroxybenzoic acid (3,5-DHBA) (both from Sigma-Aldrich, Milan, Italy) were added into the cell culture of all experiments at final concentrations of 20 mM and 150 μM, respectively, for 24, 48, and 72 h.

### Clonogenic Assay and Surviving Fraction

The clonogenic assay was performed with the Operetta High-Content Screening (HCS) System (PerkinElmer, Waltham, MA, USA) and the surviving fractions obtained as previously described (16, 17). Briefly, colony assays were performed by seeding cells in 6-well plates at a low density (2,000 cells/well) and allowing growth for 10 days. Colonies were fixed and incubated with 0.05% crystal violet diluted in 20% ethanol for 30 min at room temperature. They were then quantified with the Operetta HCS System (Perkin-Elmer) and the surviving fraction obtained normalizing the counted colonies over the total plated cells, which was expressed as the percentage of control assumed as 100%. Each experiment was performed in quadruplicate.

### Real-Time Monitoring of Cell Proliferation

xCELLigence experiments were performed using the Real-Time Cell Analysis (RTCA) dual plate (DP) instrument according to the manufacturers’ instructions (Roche Applied Science, Mannheim, Germany, and ACEA Biosciences, San Diego, CA, USA). The RTCA DP Instrument includes three main components: i) RTCA DP analyzer, which is placed inside a humidified incubator maintained at 37°C and 5% CO_2_; ii) RTCA control unit with the preinstalled RTCA software; and iii) E-Plate 16 for the proliferation assay. Firstly, the optimal seeding number was determined by cell titration and growth experiments. After seeding the optimal cell number (3,000 cells/well), the cells were treated and automatically monitored every 15 min for 24 h. The optimal cell number was determined in a preliminary set of experiments (data not shown) to obtain a significant cell index value and a constant cell growth during the entire duration of the experiment.

### Cell Migration

Cell migration was examined by employing the wound healing assay. Briefly, the cells were seeded in 24-well dishes and cultured until confluence. Then, they were treated with vehicle, lactate, or 3,5-DHBA, scraped with a 200-μl micropipette tip, and monitored at 0, 24, and 48 h. The uncovered wound area was measured and quantified at different intervals with ImageJ v1.37 (NIH, Bethesda, MD, USA).

### Immunoblotting

Briefly, for Western blot analysis, 30 μg of protein was loaded onto a 12% polyacrylamide gel, MiniPROTEAN^®^ TGXTM (Bio-Rad, Milan, Italy), followed by electrotransfer to a nitrocellulose membrane, TransBlot^®^ TurboTM, using TransBlot^®^ SE Semi-Dry Transfer Cell (both from Bio-Rad, Milan, Italy) (18). Subsequently, the membrane was blocked in Odyssey Blocking Buffer (Licor, Milan, Italy) for 1 h at room temperature. After blocking, the membrane was washed three times in phosphate-buffered saline (PBS) for 5 min and incubated with primary antibodies against MCT1 (1:1,000; AB90582), MCT4 (1:1,000; AB74109), β-catenin (1:500; AB16051), E-cadherin (1:500; AB76055, all from Abcam, Cambridge, UK), and β-actin (1:1,000; anti-mouse, cat. no. 4967S; Cell Signalling Technology, Milan, Italy) overnight at 4°C. The next day, the membranes were washed three times in PBS for 5 min and incubated with infrared anti-mouse IRDye800CW (1:5,000) and anti-rabbit IRDye700CW secondary antibodies (1:5,000) in PBS/0.5% Tween-20 for 1 h at room temperature. All antibodies were diluted in Odyssey Blocking Buffer. The blots were visualized using Odyssey Infrared Imaging Scanner (Licor, Milan, Italy), and the protein levels were quantified by densitometric analysis. Data were normalized to the expression of β-actin.

### Real-Time RT-PCR for Gene Expression Analysis

RNA was extracted using Trizol^®^ reagent (Invitrogen, Carlsbad, CA, USA) (19). First-strand complementary DNA (cDNA) was then synthesized with a reverse transcription reagent from Applied Biosystems (Foster City, CA, USA). Quantitative real-time PCR (qRT-PCR) was performed in StepOne Fast Real-Time PCR System (Applied Biosystems) using the SYBR Green PCR MasterMix (Life Technologies, Monza, Italy) (20). The specific PCR products were detected with SYBR Green fluorescence. The relative messenger RNA (mRNA) expression level was calculated by the threshold cycle (*C*_t_) value of each PCR product and normalized with that of actin using a comparative 2^−ΔΔ^*^C^*^t^ method. The sequences of the primers used are presented in **Table 1**.

**Table 1:** Genes of interest.

Genes	Forward primer (5′⟶3′)	Reverse primer (5′⟶3′)
*PGC1α*	ATGAAGGGTACTTTTCTGCCCC	GGTCTTCACCAACCAGAGCA
*SIRT1*	AGGCCACGGATAGGTCCATA	GTGGAGGTATTGTTTCCGGC
*TFAM*	CCGAGGTGGTTTTCATCTGT	AGTCTTCAGCTTTTCCTGCG
*ND4*	CCAGTGGAATGCCTTGCCTA	TTGATCGCGGTGAGATTCCC
*CyB*	ACGAGCCACCGAAACAGAAT	ACGATTTTCGCCAGTCACCT
*COX II*	ACGACCTCGATGTTGGATCA	ATCATTTACGGGGGAAGGCG
*COX IV*	GCGGCAGAATGTTGGCTAC	AGACAGGTGCTTGACATGGG
*ATP synthase*	CCGCCTTCCGCGGTATAATC	ATGTACGCGGGCAATACCAT
*MCT1*	TGTTGTTGCAAATGGAGTGT	AAGTCGATAATTGATGCCCATGCCAA
*MCT4*	TATCCAGATCTACCTCACCAC	GGCCTGGCAAAGATGTCGATGA
*HCAR1*	TTCGTATTTGGTGGCAGGCA	TTTCGAGGGGTCCAGGTACA
*β-Actin*	CCTTTGCCGATCCGCCG	AACATGATCTGGGTCATCTTCTCGC

### Zebrafish Model

Adult zebrafish (*Danio rerio*) were housed in the Model Organism Facility—Center for Integrative Biology (CIBIO), University of Trento, and maintained under standard conditions (21). All zebrafish studies were performed according to European and Italian laws (D.Lgs. 26/2014, authorization 148/2018-PR to M.C. Mione). Fishes with somatic and germline expression of oncogenic HRAS were generated as described (22, 23). The following zebrafish transgenic lines were used in the course of this study: *Et(zic4:Gal4TA4, UAS:mCherry)_hzm5_*, called zic:Gal4 (22), and *Tg(UAS:eGFP-HRAS_G12V)_io006_*, called UAS : RAS (23). The characterization of the GBM model is described in detail in Mayrhofer et al. (22).

### Gene Expression Analysis

Analysis of the expressions of the genes involved in glycolysis in zebrafish brain tumors was performed on previously generated data (GSE74754; https://www.ncbi.nlm.nih.gov/geo/query/acc.cgi?acc=GSE74754). A heatmap was generated using the web application Heatmapper (http://www.heatmapper.ca/).

For the gene expression analysis of further samples, total RNA was extracted from larval heads and brains/tumors with the TRIzol reagent (Invitrogen). Total RNA was cleaned up using the RNeasy Mini Kit (Qiagen, Hilden, Germany) following the manufacturer’s instructions and treated twice with DNase I (1 U/μg RNA; Qiagen). The RNA concentration was quantified using NanoDrop 2000 (Thermo Fisher, Waltham, MA, USA), and VILO SuperScript Kit (Thermo Fisher) was used for first-strand cDNA synthesis performed according to the manufacturer’s protocol. qRT-PCR analysis was performed using the qPCRBIO SyGreen Mix (Resnova–PCR Biosystem, Rome, Italy) following a standard amplification protocol. The primers used were as follows: for zebrafish *mct1*: forward 5′-GTCACCATTGTGGAATGTGC-3′ and reverse 5′-TCATCATAGATATCGTTGAGTCGTC-3′; for zebrafish *hcar1*: forward 5′-CATCGTCATCTACTGCTCCAC-3′ and reverse 5′-GCTAACACAAACCGCACA-3′; and for zebrafish *rps11* (housekeeping): forward 5′-ACAGAAATGCCCCTTCACTG-3′ and reverse: 5′-GCCTCTTCTCAAAACGGTTG-3′. RT-PCR was performed with a CFX96 Real-Time PCR Detection System (Bio-Rad) machine. Quantitative PCR analysis was performed with Microsoft Excel and GraphPad Prism. In all cases, each PCR was performed with triplicate samples and repeated with at least two independent samples.

### Immunofluorescence in Zebrafish

Adult zebrafish resulting from crosses between zic:Gal4 and UAS: RAS, or from the somatic expression of UAS : RAS (22), were screened under a fluorescent stereomicroscope for the presence of GFP-HRAS^G12V^ brain masses. Positive fish (over 90% of screened fish) were sacrificed by an overdose of MS222 and their brains removed, fixed, and sectioned as previously described (22).

The sections were then washed in PBS (pH 7.4) and incubated with primary antibodies diluted in PBS containing 5% normal goat serum and 0.1% Triton X-100 at 4°C overnight. The antibodies used and their dilutions were as follows: MCT1 (1:100), HCAR1 (1:100), and phospho-histone 3 (1:1,000; all from Abcam, Cambridge, UK). A secondary antibody conjugated with Alexa 546 (1:250; Abcam) was used for 2 h at room temperature, and the nuclei were counterstained with DAPI. Images were acquired using an inverted Leica TSP8 confocal microscope. For whole-mount immunofluorescence of 5-day post-fertilization (dpf) zebrafish, the larvae of the zic:Gal4 line (controls) or zic:Gal4 × UAS : RAS line (tumor) were treated with 20 mM lactate or 10 mM AZ3965 in 1% dimethyl sulfoxide (DMSO) in E3, or with 1% DMSO alone. Solutions with the drugs were changed every day starting at 1 dpf until 5 dpf, when the larvae were culled by anesthetic overdose, fixed in 4% paraformaldehyde (PFA) for 2–12 h at 4 C, their brains carefully removed under a stereomicroscope and processed with Ph3 antibody, and diluted 1:1,000 in 5% normal goat serum (NGS) and 0.5% Triton X100 in PBS overnight. A secondary antibody conjugated with Alexa 546 was used for 6 h at room temperature. Images were acquired using an inverted Leica TSP8 confocal microscope after equilibrating the brains in 100% glycerol.

### Seahorse on Zebrafish

For Seahorse analysis, tumors from adult fish or control brains were dissociated with a pipette tip in the assay medium provided by the manufacturer, passed through a 40-mM sieve, and counted. A total of 50,000 cells were seeded on poly-d-lysin-coated Seahorse XFP plates and incubated for 20 min in the absence of CO_2_ before adding medium up to a final volume of 180 μl. The XF Mito Stress Test kit including oligomycin, carbonyl cyanide *p*-trifluoromethoxyphenylhydrazone (FCCP), rotenone A, and UK5099 was obtained from Seahorse Bioscience, Inc. (Billerica, MA, USA). The XFp cell culture plates, sensor cartridges, and XF base medium were also purchased from Seahorse Bioscience, Inc. The Agilent Seahorse XFp Sensor Cartridge was hydrated in the Agilent Seahorse XF Calibrant at 28°C in a non-CO_2_ incubator overnight. Zebrafish brain tumor cells were plated in the Agilent Seahorse XFp Cell Culture Miniplate at the desired density (50,000 per well) using the appropriate cell culture growth medium. We added 1× PBS to the chambers to prevent evaporation of the culture medium. Within 1 h from plating, the Agilent Seahorse XFp Cell Culture Miniplate was placed into a 28°C non-CO_2_ incubator for 1 h prior to the assay (24).

For the Mito Stress test, the assay medium was prepared by supplementing the Agilent Seahorse XF base medium with 1 mM pyruvate, 2 mM glutamine, and 10 mM glucose, bringing the pH to 7.4 with 0.1 N NaOH. The cells were placed in a 28°C incubator without CO_2_. Oligomycin (final concentration, 2.5 μM), FCCP (final concentration, 2 μM), and rotenone A (final concentration, 0.5 μM) were diluted in the assay medium following the user guide for the Agilent Seahorse XFp Mito Stress test and then loaded into ports B, C, and D, respectively. In port A, we placed either the assay medium, lactate (final concentration, 20 mM), AZ3965 (final concentration, 10 μM), or UK5099 (final concentration, 2 μM). The machine was calibrated at 28°C, and the assay was performed using the acute Mito Stress test assay protocol as suggested by the manufacturer (Seahorse Bioscience, Inc., Billerica, MA, USA). The oxygen consumption rate (OCR) was measured under basal conditions and after injection of the assay medium (control), lactate, UK5099, or AZ3965, followed by the sequential addition of oligomycin, FCCP, and rotenone/antimycin A. All Seahorse data (at least 3 biological replicates) were normalized to the total number of cells and counted by nuclear DAPI staining following the assay. The XF reports of the Mito Stress data were analyzed with the freeware Wave and exported to Excel and Prism for further analysis and visualization.

### Glioblastoma Biopsies

Formalin-fixed and paraffin-embedded (FFPE) tissue specimens from 10 patients affected by GBM were obtained from the surgical pathology files at the Anatomic Pathology, Department G.F. Ingrassia, University of Catania, Catania, Italy. Multiple sections (at least 5) were obtained from FFPE tissue specimens. Due to the retrospective nature of the study, no written informed consent was obtained from the patients. The study included 6 male and 4 female patients (mean age = 61 years, range = 41–81 years). The study was conducted according to the guidelines of the Declaration of Helsinki and approved by the Catania 1 Ethics Committee, Catania, Italy (protocol code: 166/2015/PO; 17/12/2015). According to the WHO criteria, the histological diagnosis of GBM was rendered in the presence of the following morphological criteria: i) high-grade glioma with astrocytic morphology; ii) diffuse growth pattern; and iii) foci of necrosis and/or microvascular proliferation.

### Immunohistochemical Analysis

Sections were processed as previously described (25). Thereafter, they were incubated overnight at 4°C with rabbit polyclonal anti-MCT1 antibody (Sigma, Milan, Italy), ready-to-use PBS (Sigma, Milan, Italy), and MIB-1, a monoclonal antibody directed against the Ki67 antigen (M7240; Dako Corporation, Glostrup, Denmark), and diluted 1:75 in PBS. The secondary antibody, biotinylated anti-rabbit antibody, was applied for 30 min at room temperature, followed by the avidin–biotin–peroxidase complex (Vector Laboratories, Burlingame, CA, USA) for a further 30 min at room temperature. The immunoreaction was visualized by incubating the sections for 4 min in 0.1% 3,3′-diaminobenzidine (DAB) and 0.02% hydrogen peroxide solution (DAB substrate kit; Vector Laboratories, Burlingame, CA, USA). The sections were lightly counterstained with Mayer’s hematoxylin (Histolab Products AB, Göteborg, Sweden) mounted in glycerol vinyl alcohol (GVA) mounting medium (Zymed Laboratories, San Francisco, CA, USA) and observed with a Zeiss Axioplan light microscope (Carl Zeiss, Oberkochen, Germany). MCT1 staining (both nuclear and cytoplasmic) was semi-quantitatively evaluated according to a 0 to 3 scale of intensity of staining (IS) and to the percentage of positively stained cells [extent score (ES) on a five-tier system: <5%, 5%–30%, 31%–50%, 51%–75%, and >75%].

The immunohistochemical expression of MIB-1 was assessed as low if positive in less than 50% of neoplastic cells and as high if positive in more than 50% of neoplastic cells.

### Human Gene Expression

#### Dataset Selection

The NCBI Gene Expression Omnibus (GEO) database (http://www.ncbi.nlm.nih.gov/geo/) (26) was used to select transcriptome datasets of interest. The mesh terms “human,” “glioblastoma,” and “tumor grade” were used to identify the datasets. We sorted the datasets by the number of samples (from high to low), age, and sex of the participants and by the clinical data made available by the authors. We selected the GSE108474 dataset (27) over the others available for the number of subjects recruited (541), for the availability of clinical data (tumor staging), and for the variety of tumors analyzed (GBM, oligodendrocytoma, astrocytoma, and normal subjects).

### Data Processing, Experimental Design, and Statistics

For statistical analyses, a two-tailed unpaired Student’s *t*-test was used for the comparison of two groups. For the comparison of three or more groups, one-way analysis of variance (ANOVA) followed by Bonferroni *post-hoc* test for multiple comparisons was used. Data are presented as the mean ± SEM of biological replicates. Data analysis was performed using GraphPad Prism software, version 5.00. A value of *p* < 0.05 was considered statistically significant (the symbols used to indicate statistical differences are described in the figure legends).

To process and identify significant differentially expressed genes (SDEGs) within the datasets, we used the MultiExperiment Viewer (MeV) software [The Institute for Genomic Research (TIGR), J. Craig Venter Institute, La Jolla, CA, USA]. In cases where multiple gene probes have insisted on the same NCBI GeneID, we used those with the highest variance. For GSE108474 (**Table 2**), we performed statistical analysis with GEO2R, applying the Benjamini and Hochberg procedure (false discovery rate) (28–30). **Table 2** presents the results of sample detection from the GSE dataset and the significant differences between groups assessed using ordinary one-way ANOVA; correction with Tukey’s multiple comparison test was also performed to compare the data between all groups. Correlations were determined using Pearson’s correlation. All tests were two-sided, and significance was determined at an adjusted *p*-value of 0.05. The dataset selected was transformed for the analysis of *Z*-score intensity signal. The *Z*-scores were calculated by taking the ratio of the weighted mean difference to the combined standard deviation according to Box and Tiao (31). The application of a classical method of data normalization, *Z*-score transformation, provides a way of standardizing the data across a wide range of experiments and allows the comparison of microarray data independent of the original hybridization intensities. The *Z*-score is considered a reliable procedure for this type of analysis and can be considered a state-of-the-art method, as demonstrated by numerous research works (32–43). The efficiency of each biomarker across the different tumor grades was assessed by analysis of the receiver operating characteristic (ROC) curves (44–46). The ROC curves analyzed the brain biopsies of healthy subjects (non-tumor, NT) *vs*. GBM patients, astrocytoma *vs*. GBM, and oligodendroglioma *vs*. GBM. The area under the ROC curve (AUC) and its 95% confidence interval (95% CI) indicated diagnostic efficiency. The accuracy of the test with the percent errors were reported (47).

**Table 2:** Samples selected from the GSE108474 dataset.

Disease type	Number	Grade
NT	28	Negative
Astrocytoma	148	G2 = 65; G3 = 58; Na = 25
Oligodendrocytoma	67	G2 = 30; G3 = 23; Na = 14
Glioblastoma	221	G4 = 130; Na = 91

G, tumor grade; Na, not assigned; NT, non-tumor

## Results

### Lactate Induces Glioblastoma Cell Proliferation and Migration *via HCAR1* and *MCT1*

We first analyzed the effects of lactate on 3 human GBM cell lines (i.e., U-87 MG, A-172, and U-251 MG) by performing a clonogenic assay on lactate-exposed cells (**Supplementary Figure S1**). It was observed that lactate induced an increase of about 2-fold in both the number (78.3 ± 9.0 control vs. 151.0 ± 17.1 lactate) and area (123.2 ± 8.2 control vs. 215.0 ± 30.4 lactate) of colonies of U-87 MG cells (**Supplementary Figure S1**). Interestingly, analysis of clonogenicity on A-172 revealed that lactate reduced the total number of colonies (35.7 ± 0.3 control vs. 21.0 ± 1.2 lactate) (**Supplementary Figure S1**), but dramatically affected the area of colonies, which was increased more than 4-fold compared to control cultures (731.3 ± 0.5 control vs. 3470.8 ± 30.3 lactate) (**Supplementary Figure S1**). We also repeated our analysis on U-251 MG cells, which showed a similar response observed in U-87 MG cells, with a significant increase in the total number of colonies (26.5 ± 0.9 control vs. 38.0 ± 3.6 lactate) and mean colony area (700.0 ± 7.1 control vs. 1409.4 ± 28.0 lactate) (**Supplementary Figure S1**).

**Figure 1:**
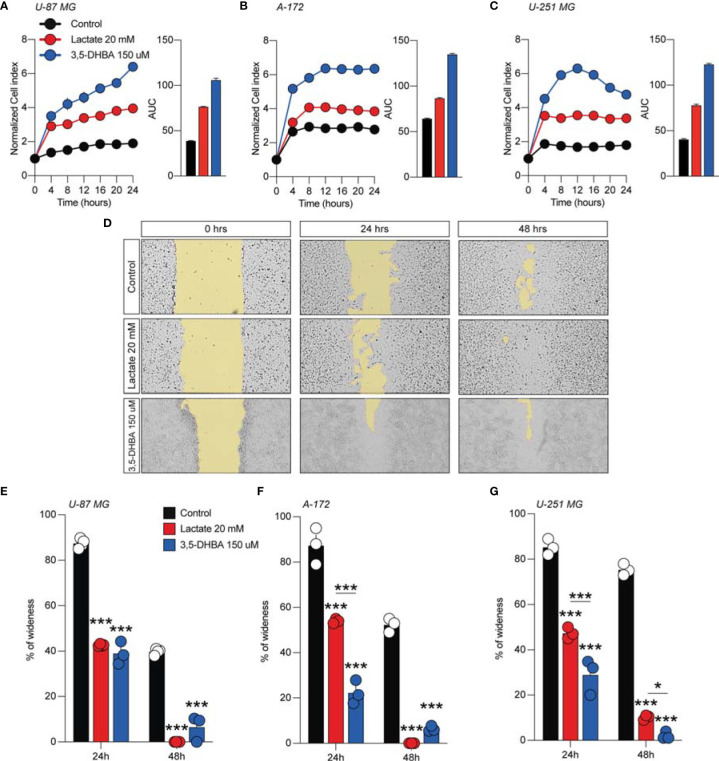
Lactate and 3,5-dihydroxybenzoic acid (3,5-DHBA) promoted glioblastoma cell proliferation and migration. **(A–C)** Real-time cell proliferation monitoring in U-87 MG cells **(A)**, A-172 cells **(B)**, and U-251 cells **(C)** using the xCELLigence system following treatments with lactate (20 mM) and 3,5-DHBA (150 μM). The cell index values were normalized at the time of pharmacological treatments in order to obtain a normalized cell index. *Each line* expresses the average of four different experiments. **(E–G)** Analysis of human glioblastoma cell migration in U-87 MG cells **(E)**, A-172 cells **(D–F)**, and U-251 MG cells **(G)** with the wound healing assay following treatments with lactate (20 mM) and 3,5-DHBA (150 μM). The figures presented are representative of at least three independent experiments (mean ± SEM). *P*-values <0.05 were considered as statistically significant (*p < 0.05; ***p < 0.001).

We then subsequently compared the effect of increased levels of extracellular lactate with the selective stimulation of the lactate receptor HCAR1 mediated by 3,5-DHBA. We observed in all tested cells a significant increase of the normalized cell index after lactate exposure (**Figures 1A**–**C**), confirmed by an increase of the total AUCs for U-87 MG (76.5 ± 0.4 lactate vs. 38.9 ± 0.2 control), A-172 (86.6 ± 0.8 lactate vs. 64.4 ± 0.4 control), and U-251 MG (78.1 ± 1.1 lactate vs. 40.5 ± 0.8 control) (**Figures 1A**–**C**, respectively). 3,5-DHBA stimulation was also able to induce similar effects on cell proliferation on the U-87 MG, A-172, and U-251 MG cell lines, showing increased normalized cell indices in all tested cell lines confirmed by an increase of the total AUCs for U-87 MG (106.3 ± 2.4), A-172 (134.6 ± 1.1), and U-251 MG (122.9 ± 1.3) (**Figures 1A**–**C**, respectively). We then tested whether lactate affects the cell migration of GBM cells. Interestingly, we observed a reduced percentage of wideness in the scratch assay at 24 and 48 h in all tested cell lines (**Figures 1D**–**G**). We also confirmed the effects of HCAR1 stimulation through 3,5-DHBA on cell migration, finding a significantly reduced percentage of wideness in the scratch assay at 48 h in all tested cell lines (**Figures 1D**–**G**).

**Figure 2:**
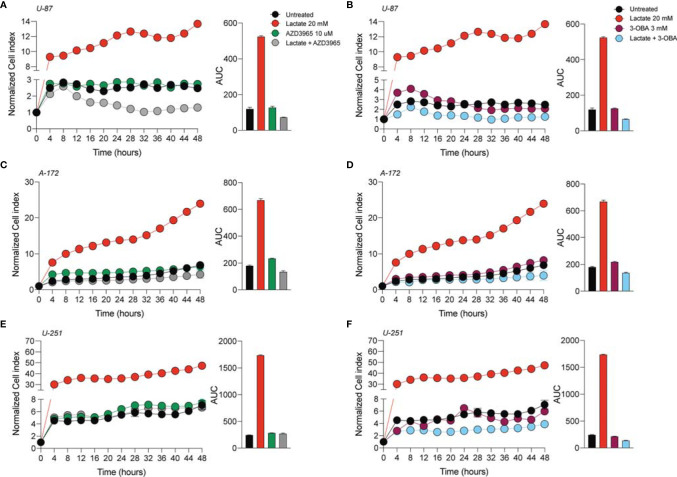
AZD3965 and 3-OBA reduced glioblastoma cell proliferation. **(A–F)** Real-time cell proliferation monitoring in U-87 MG cells **(A, B)**, A-172 cells **(C, D)**, and U-251 cells **(E, F)** using the xCELLigence system following treatments with lactate (20 mM), AZD3965 (10 μM), and 3-OBA (3 mM). The cell index values were normalized at the time of pharmacological treatments in order to obtain a normalized cell index. *Each line* expresses the average of four different experiments.

In order to verify whether lactate metabolism was involved in these effects on GBM cell proliferation and migration, we analyzed the effect of the MCT1 inhibitor (AZD3965, 10 μM) and the HCAR1 antagonist (3-OBA, 3 mM). Interestingly, our results showed that treatments of both AZD3965 and 3-OBA alone had no effect on cell proliferation, as indicated by the normalized cell index and AUC values compared to untreated control cells in all three tested cell lines (**Figures 2A**–**F**). Moreover, the U-87 MG cell line co-treated with lactate/AZD3965 showed significant decreases in the cell index (**Figure 2A**) and the AUC (73.14 ± 1.2 lactate/AZD3965 vs. 120.3 ± 8.6 untreated) (**Figure 2A**), while the A-172 and U-251 MG cell lines showed no significant effects (**Figures 2B, E**). However, the co-treatment of lactate/3-OBA showed decreased normalized cell indices for all three cell lines tested (**Figures 2B, D, F**), as confirmed by the decreased total AUCs of U-87 MG (65.1 ± 1.9 lactate/3-OBA vs. 120.3 ± 8.6 untreated), A-172 (137.0 ± 5.1 lactate/3-OBA vs. 181. ± 3.5 untreated), and U-251 MG (139.4 ± 3.9 lactate/3-OBA vs. 245.2 ± 4.8 untreated) (**Figures 2B, D, F**, respectively). We then evaluated whether MCT1 inhibition and HCAR1 antagonism affect the cell migration and surviving fraction (**Supplementary Figures S2, S3**). The results showed that both co-treatments (lactate/AZD3965 and lactate/3-OBA) resulted in a significant increase in the percentage of wideness in the scratch assay compared to the lactate and untreated control cells in the U-87 MG cell line (68.0 ± 2.1 lactate/AZD3965, 61.5 ± 4.5 lactate/3-OBA vs. 0.0 ± 0.0 lactate, vs. 25.75 ± 8.1 untreated) (**Supplementary Figures S2B, E**) and the A-172 cell line (47.3 ± 5.3 lactate/AZD3965, 87.1 ± 4.9 lactate/3-OBA vs. 0.0 ± 0.0 lactate, vs. 22.3 ± 2.3 untreated) (**Supplementary Figures S2A, C, F**) cell lines, as well as a significant increase in the percentage of wideness in the scratch assay compared to lactate in the U-251 MG cell line (58.7 ± 3.2 lactate/AZD3965, 64.7 ± 5.5 lactate/3-OBA vs. 26.5 ± 5.0 lactate) (**Supplementary Figures S2D, G**). The same results were obtained in the surviving fraction assay (**Supplementary Figure S3**).

### *HCAR1* Targeting Increases the Expression Levels of *MCT1* and *MCT4*

In an effort to link lactate, as a positive modulator of cell proliferation and migration, to the underlying molecular mechanisms activated in GBM cell lines, we performed Western blot analysis for the lactate transporters MCT1 and MCT4 and for β-catenin and E-cadherin on the control and lactate-treated U-87 MG, A-172, and U-251 MG cells.

**Figure 3:**
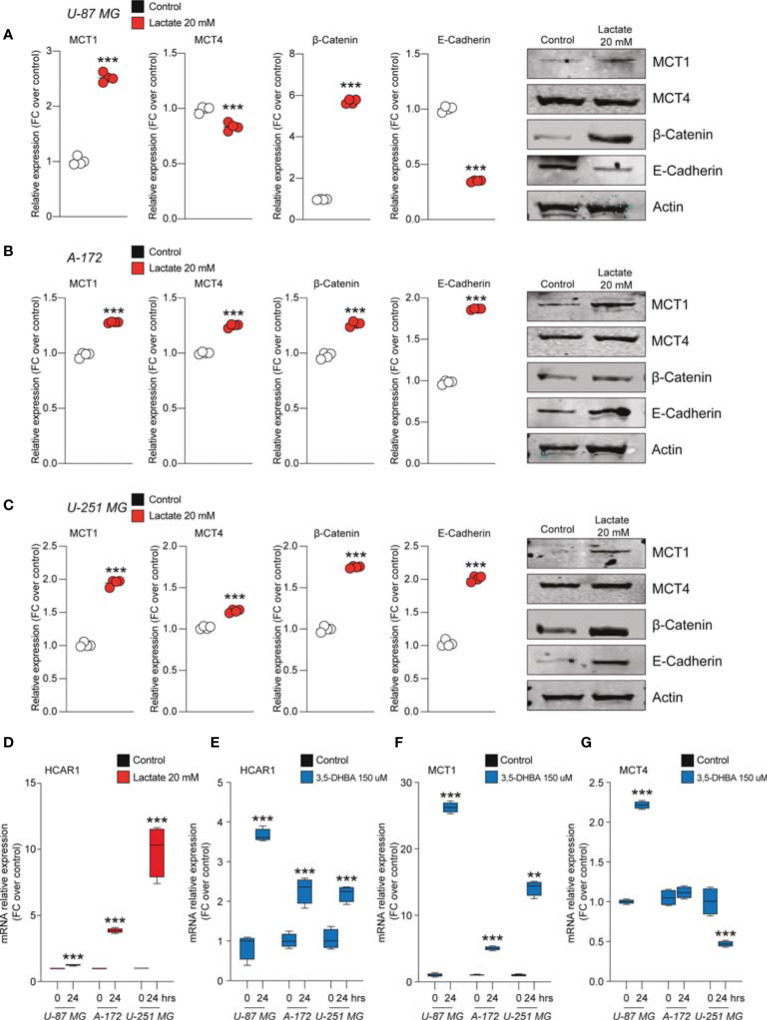
Lactate regulated the expression of monocarboxylate transporters (MCTs) and epithelial–mesenchymal transition (EMT) markers in glioblastoma cells. **(A–C)** Protein expressions of MCT1, MCT4, β-catenin, and E-cadherin in U-87 MG cells **(A)**, A-172 cells **(B)**, and U-251 MG cells **(C)** following 72 h of lactate (20 mM) treatment. The figures presented are representative of at least four independent experiments, and values represent the mean ± SEM of experiments performed in quadruplicate. **(D)** *HCAR1* gene expression in U-87 MG, A-172, and U-251 MG cells following 24 h of lactate (20 mM) treatment. **(E–G)** Gene expressions of *HCAR1* **(E)**, *MCT1* **(F)**, and *MCT4* **(G)** in U-87 MG, A172, and U-251 MG cells following 24 h of 3,5-dihydroxybenzoic acid (3,5-DHBA, 150 μM) treatment. Values represent the mean ± SEM of experiments performed in quadruplicate. *P*-values <0.05 were considered as statistically significant (**p < 0.01; ***p < 0.001 *vs*. untreated).

We found that U-87 MG cells responded to increased levels of extracellular lactate by increasing the levels of the MCT1 transporter about 2.5-fold compared to control cultures and slightly, but significantly, reducing the expression level of MCT4 (**Figure 3A**).

Importantly, the protein levels of β-catenin were found to be significantly increase about 6-fold in lactate-exposed U-87 MG cells, and such a modulation was coupled with reduced expression levels of E-cadherin (**Figure 3A**). Notably, analysis of A-172 and U-251 MG cells exposed to increased extracellular lactate levels revealed some differences in the cellular responses compared to U-87 MG cells. Indeed, it was confirmed that exposure to lactate increased the expression levels of MCT1 and β-catenin in both A-172 (**Figure 3B**) and U-251 MG (**Figure 3C**) cells, but showed that the response of both cell lines to lactate also induced significantly higher MCT4, an increase of about 1.2-fold in both, and E-cadherin expression levels (**Figures 3B, C**).

Given the evidence on cellular modulation exerted by increased extracellular levels of lactate, we sought to link the molecular mechanisms underlying these phenomena with the activation of the lactate receptor HCAR1. We first investigated the mRNA expression levels of HCAR1 on the U-87 MG, A-172, and U-251 MG cell lines after exposure to lactate, which revealed a significant increase of the mRNA levels of HCAR1 in all tested cells at 24 h (**Figure 3D**). We then evaluated the effects of 3,5-DHBA, confirming a significant increase of the mRNA levels of HCAR1 in all tested cell lines 24 h after 3,5-DHBA incubation (**Figure 3E**).

To determine whether selective stimulation of HCAR1 was able to increase the expressions of the lactate transporters MCT1 and MCT4, we also checked the mRNA expression levels of these transporters, finding that 3,5-DHBA stimulation was able to significantly increase MCT1 expression about 25-, 5-, and 13-fold in U-87 MG, A-172, and U-251 MG cells, respectively (**Figure 3F**). Such pieces of evidence were coupled with contrasting data on the other tested transporter, MCT4. Indeed, we observed that U-87 MG cells responded to 3,5-DHBA stimulation by increasing the mRNA expression of MCT4 about 2-fold (**Figure 3G**), but A-172 cells showed no significant changes in MCT4 expression, while U-251 MG cells showed a significant reduction of MCT4 expression upon treatment with 3,5-DHBA compared to untreated cells (**Figure 3G**).

### Lactate Stimulation on Glioblastoma Cell Lines Increases Mitochondrial Fitness and Energy Metabolism *via HCAR1*

**Figure 4:**
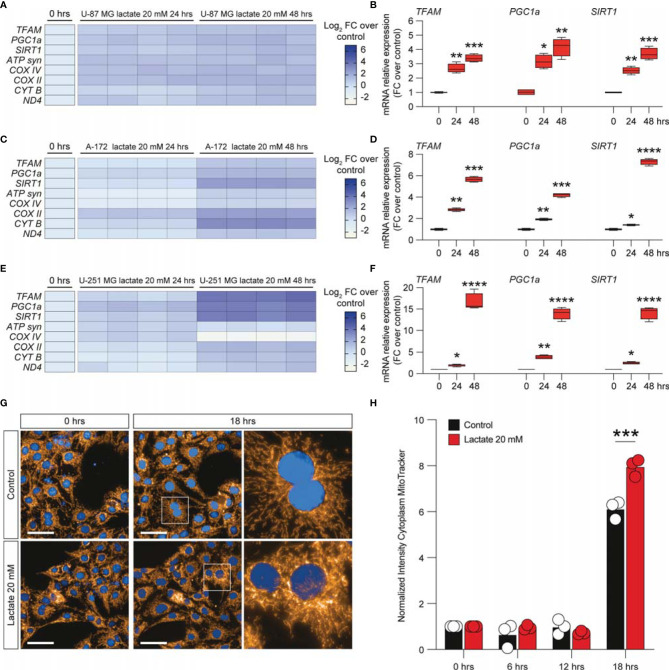
Lactate promoted the upregulation of mitochondrial activity gene expressions in glioblastoma cells. **(A–F)** Effect of lactate (20 mM) on mitochondrial biogenesis and *OXPHOS* gene expression in U-87 MG cells **(A, B)**, A-172 cells **(C, D)**, and U-251 MG cells **(E, F)** following 24 and 48 h of treatment. **(G, H)** Computerized analysis of the MitoTracker fluorescence intensity in the control *versus* lactate 18 h after treatment. The figures presented are representative of at least three independent experiments. Values represent the mean ± SEM of experiments performed in quadruplicate. *P*-values <0.05 were considered as statistically significant (*p < 0.05; **p < 0.01; ***p < 0.001 ; ****p < 0.0001 *vs*. untreated).

**Figure 5:**
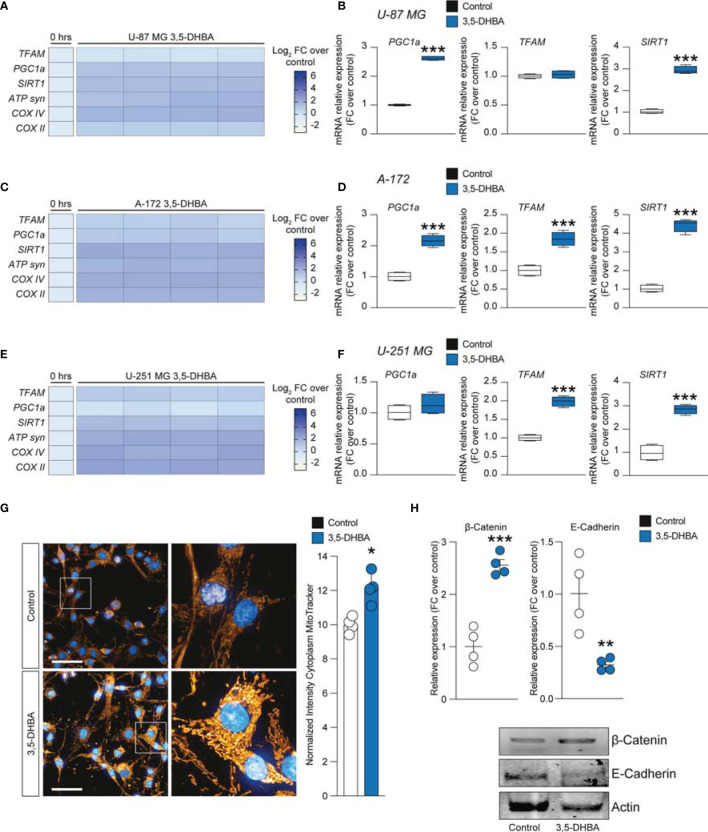
*HCAR1* selective stimulation promoted the upregulation of mitochondrial activity gene expressions and regulated the protein expressions of the epithelial–mesenchymal transition (EMT) markers in glioblastoma cells. **(A–F)** Effect of 3,5-dihydroxybenzoic acid (3,5-DHBA, 150 μM) on mitochondrial biogenesis and *OXPHOS* gene expression in U-87 MG cells **(A, B)**, A-172 cells **(C, D)**, and U-251 MG cells **(E, F)** following 24 h of treatment. **(G)** Computerized analysis of the MitoTracker fluorescence intensity on the control *versus* lactate 18 h after treatment. The figures presented are representative of at least three independent experiments. **(H)** Protein expressions of β-catenin and E-cadherin in A-172 cells following 72 h of *HCAR1* stimulation. The figures presented are representative of at least four independent experiments, and values represent the mean ± SEM of experiments performed in quadruplicate. *P*-values <0.05 were considered as statistically significant (*p < 0.05; **p < 0.01; ***p < 0.001 *vs*. untreated).

**Figure 6:**
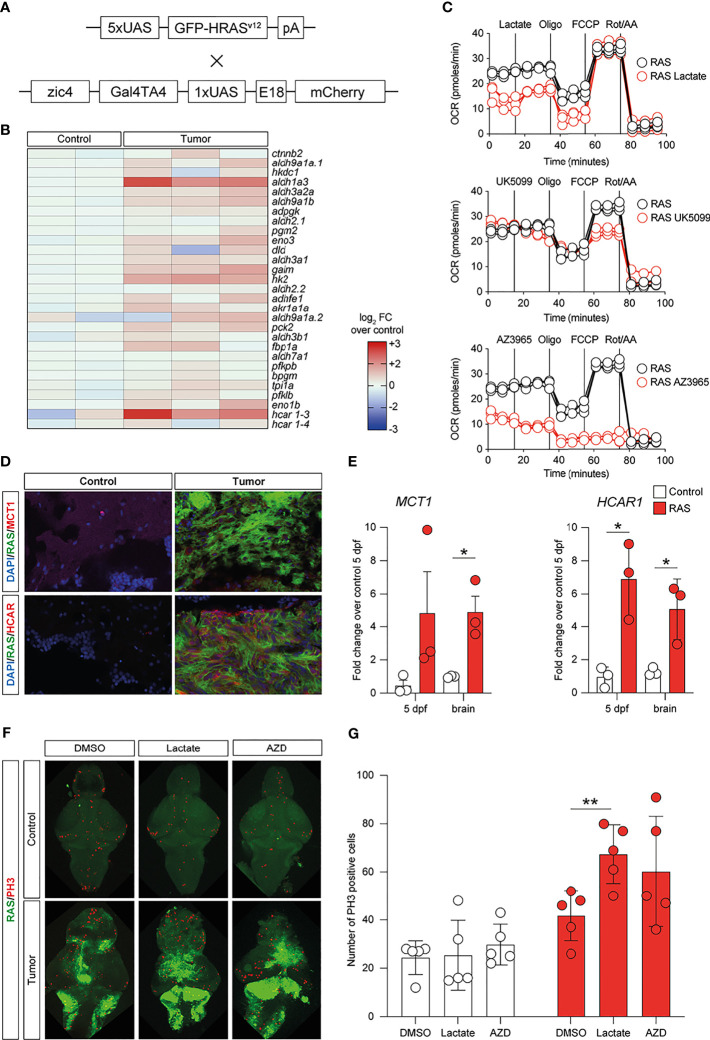
Metabolic changes in a zebrafish model of glioblastoma (GBM) led to increased glycolysis and lactate transport and sensing. **(A)** Schematic representation of the genetic components of the zebrafish GBM model (Mayrhofer et al., 2017). **(B)** Increased expression of several members of the glycolytic pathway in GBM. Heatmap representing 29 glycolysis genes and their relative expression levels. **(C)** Analysis of mitochondrial metabolism (acute Mito Stress test) of tumor cells with the Seahorse XP technology. Each experiment was performed in triplicate and normalized to the number of cells. **(D)** Increased levels of *HCAR1* in tumors *vs*. control as visualized by immunofluorescence. Staining as detailed in the figures, which are representative of at least three different experiments. **(E)** Gene expression analysis through quantitative PCR (qPCR) expressed as fold changes compared to controls, at 5 days post-fertilization and in adult tumors. Values represent the mean ± SEM of experiments performed in triplicate. *P*-values <0.05 were considered as statistically significant (*p < 0.05 *vs*. controls). **(F)** Whole-mount immunofluorescence of Ph3 proliferating cells in controls and in HRAS-overexpressing larvae treated or not with 20 mM lactate. Green fluorescence represents tumoral cells expressing eGFP-HRASG12V. **(G)** Number of proliferating cells in the brains treated as indicated.

**Figure 7:**
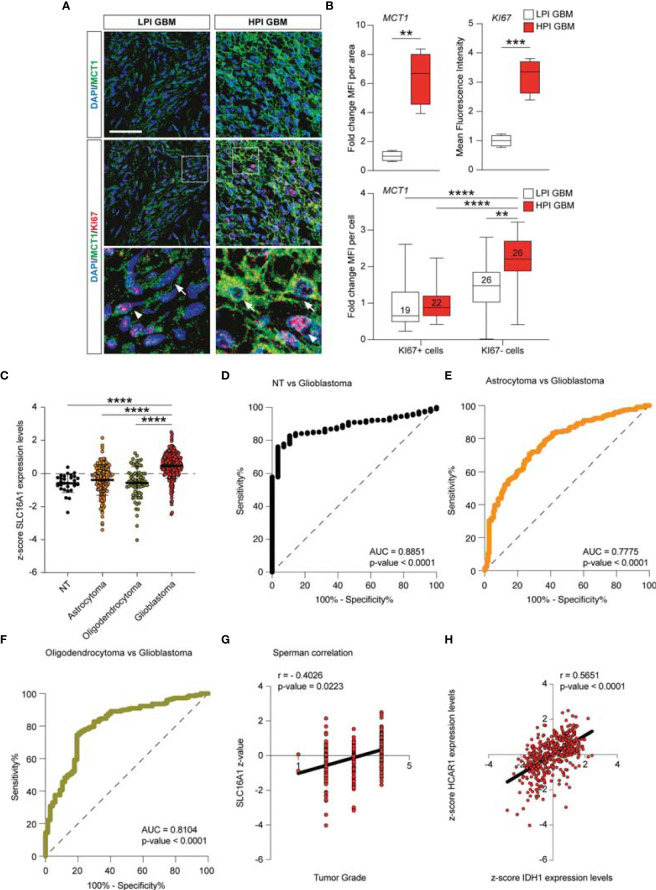
*MCT1* expression analysis from the human brain tumor GSE108474 dataset. (**A**) Analysis of the MCT1 gene expression in brain biopsies of patients with astrocytoma, oligodendrocytoma, and glioblastoma (GBM) and in healthy subjects. (**B**) Pearson’s correlation analysis between the expression levels of MCT1 and the tumor grade of brain biopsies obtained from patients affected by main brain tumors. (**C**) Pearson’s correlation between the expression levels of MCT1 and IDH1 in brain biopsies of patients with GBM. (**D–F**) Receiver operating characteristic (ROC) analysis between the expression levels of MCT1 in the brain in healthy subjects vs. GBM patients (**D**), between GBM patients vs. astrocytoma patients (**E**), and between GMB patients vs. oligodendrocytoma patients (**F**). (**G**) Sperman correlation of SLC16A1 z-value and tumor grade. (**H**) Correlation between HCAR1 and IDH1 expression levels. Data are expressed as the mean ± SD of at least four independent experiments (**p < 0.005; ***p < 0.001; ****p < 0.0001)..

To further expand our evidence on the molecular mechanisms induced by the increase of extracellular lactate, we analyzed a panel of mRNAs of the genes involved in mitochondrial activity and energy metabolism. Our data showed that U-87 MG cells significantly increased about 4-fold the relative mRNA levels of transcription factor A, mitochondrial (TFAM), PPARG coactivator 1 alpha (PGC1a), and sirtuin 1 (SIRT1) (**Figures 4A, B**), coupled with an overall increase of ATP synthase (ATP syn), cytochrome c oxidase subunit 4 (COX IV) and COX II, mitochondrial cytochrome b (CYTB), and mitochondrial NADH-ubiquinone oxidoreductase chain 4 (ND4) (**Figure 4A**), when exposed to lactate for 24 or 48 h compared with untreated cells (**Figures 4A, B**). These observations were confirmed in the A-172 (**Figures 4C, D**) and U-251 MG (**Figures 4E, F**) cell lines. Specifically, we observed superimposable effects on A-172 compared to U-87 MG cells, where U-251 MG showed an increase of about 15-fold of TFAM, PGC1a, and SIRT1 at 48 h compared to untreated cells (**Figure 4F**), coupled with a slight reduction of COX IV mRNA at the same time point [−1.87 ± 0.1 log_2_ fold change (FC) over the control] (**Figure 4E**). We also performed a computer-assisted analysis of the MitoTracker fluorescence intensity on control versus lactate-treated cells, finding that lactate was able to significantly increase the cytoplasmic MitoTracker intensity 18 h post-treatment (**Figure 4G, H**).

To link the intracellular mediators of mitochondrial fitness with HCAR1 stimulation, we performed an analysis of the mRNA expression levels of PGC1a, TFAM, SIRT1, ATP syn, COX II, and COX IV on 3,5-DHBA-stimulated cells. Our analysis revealed that U-87 MG cells exposed to 3,5-DHBA recapitulated the molecular mRNA activation observed with lactate (**Figures 5A, B**). Indeed, all tested genes, except for TFAM, were significantly increased in cultures exposed to HCAR1 stimulation (**Figure 5B**). These data were confirmed in A-172 cells, which showed increased levels of all tested genes upon 3,5-DHBA stimulation (**Figures 5C, D**). Finally, U-251 MG showed very similar mRNA expression profiles, but we observed that HCAR1 stimulation through 3,5-DHBA did not modulate the expression of PGC1a at the tested time point in this cell line (**Figures 5E, F**).

To finally link HCAR1 stimulation with the effects on the mitochondria observed in GBM cell lines exposed to increased extracellular lactate levels, we performed a MitoTracker analysis, which demonstrated a significant increase of normalized intensity in 3,5-DHBA-stimulated cells compared to control cultures (**Figure 5G**).

Given the capability of extracellular lactate to modulate the expression levels of β-catenin and E-cadherin, we performed Western blot analysis on 3,5-DHBA-stimulated A-172 cells. Our analysis revealed that HCAR1 activation induced a significant increase in the protein expression levels of β-catenin compared to control cultures, and this phenomenon was coupled with a significant reduction of E-cadherin (**Figure 5H**), revealing that lactate may also act via additional mechanisms to induce E-cadherin not related to HCAR1 activation.

### Lactate Stimulation Modulates Metabolism and *MCT1* in the Zebrafish Model of Glioblastoma

To investigate whether lactate accumulation, resulting from increased glycolysis, may have similar effects *in vivo*, we used the zebrafish model of GBM (22) (**Figure 6A**) and analyzed the metabolic phenotypes of these tumors. Comparison of the expression levels of 29 genes encoding for enzymes and transporters involved in the glycolytic pathway acquired through RNA sequencing (GSE74754; https://www.ncbi.nlm.nih.gov/geo/query/acc.cgi?acc=GSE74754) revealed the increased expressions (log_2_ FC > 1.2, p < 0.001, or adjusted p < 0.05) of 26 out of 29 genes, with aldh1a3, hk2, and hcar1-3 being the most upregulated in tumors (**Figure 6B**). We then performed a Mito Stress test on freshly dissociated zebrafish tumor brains using the Seahorse XFp apparatus and acute injection of lactate (20 mM), UK5099 [an inhibitor of the mitochondrial pyruvate carrier (MPC), 2 μM], or AZ3695 (10 μM, to inhibit MCT1). This test confirmed that acute injection of lactate (upper panel in **Figure 6C**) had little effect on the OCR of zebrafish brain tumor cells, whereas, upon blockage of the MPC (UK5099) (middle panel in **Figure 6C**), the response of the cells at maximal respiration (after FCCP injection) was partly inhibited and, upon blockage of the MCT1 transporter (AZ3695) (lower panel in **Figure 6C**), the energy production through mitochondrial respiration was blocked, suggesting that blocking the transport of lactate has profound consequences on the ability of zebrafish GBM cells to oxidize substrates for energy production (**Figure 6C**). Staining for MCT1 and HCAR1 in sections of zebrafish brain tumors revealed an increase in the number of both MCT1+ and HCAR1+ cells (**Figure 6D**), whereas qPCR analysis of the mRNA expressions of mct1 and hcar1 revealed a significant increase in the expression for mct1 in adult tumors compared to the control brain and a significant increase in the expression of hcar1 in both adult brain tumors and in 5-dpf larvae expressing oncogenic RAS (**Figure 6E**).

Subsequently, we evaluated the effects of exposing zebrafish brain tumor cells to lactate or to the MCT1 inhibitor, AZD3965, on the proliferation rate of control brains and brains expressing oncogenic RAS using immunostaining for a mitotic marker (phosphoserine 10 on histone 3, PH3). Incubation of developing larvae from 1 to 5 dpf with 20 μM lactate induced a significant increase in proliferation in brains expressing oncogenic RAS, but not in control brains, while treatment with 10 μM AZD3965 did not affect the proliferation rate in either control or RAS-expressing brains (**Figure 6F**).

### *HCAR1* and *MCT1* Positively Correlated With Human Glioma Aggressiveness

The *MCT1* gene expression analysis obtained from the GSE108474 dataset showed that there were significant differences when the expression levels obtained from brain biopsies of GBM patients were compared to other brain tumor stages (**Figure 7**). Specifically, patients with GBM expressed significantly higher levels of the *MCT1* messenger in the brain than did patients with oligodendrocytoma (*p* < 0.0001) and astrocytoma (*p* < 0.0001) or healthy subjects (*p* < 0.0001) (**Figure 6A**). This finding was confirmed by the significantly positive correlation between the expression levels of *MCT1* and tumor grade (*r* = 0.4026, *p* = 0.0223) (**Figure 6B**). According to these results, we investigated the prognostic potential of *MCT1* expression in the progression of main brain tumors. Currently, analysis of the expression of isocitrate dehydrogenase [NADP(+)] 1 (*IDH1*) and the identification of its main mutations (e.g., R132H) were used for glioma diagnosis and prognosis (48). By carrying out Pearson’s correlation analysis between the expression levels of *MCT1* and *IDH1* in brain tumors, we highlighted that, in GBM patients, the expression levels of these two genes were significantly closely inversely correlated (*r* = −0.4163, *p* < 0.0001) (**Figure 7C**). Furthermore, in order to evaluate the potential diagnostic ability of *MCT1* gene expression to discriminate against the brain tumor stages, we performed an ROC analysis. We confirmed the diagnostic ability of *MCT1* to discriminate GBM patients from healthy subjects (AUC = 0.7558, *p* < 0.0001) (**Figure 7D**) or from patients affected by astrocytoma (AUC = 0.7775, *p* < 0.0001) (**Figure 6E**) or oligodendrocytoma (AUC = 0.8104, *p* < 0.0001) (**Figure 7F**).

## Discussion

Cell metabolism and its related intercellular signaling have been shown to be of great importance in a number of physiological and pathological processes (49). In the present study, we first evaluated the effects of lactate on three human GBM cell lines, finding that it increased both cell migration and proliferation. Such a phenomenon was linked to lactate-dependent *HCAR1* activation, as suggested by the effects of 3,5-DHBA, a selective *HCAR1* agonist.

Several authors showed that stimulation of *HCAR1* leads to the activation of cell survival signals, promoting cell proliferation *via* the inhibition of apoptosis and stimulating the secretion of several angiogenic factors in a PI3K/Akt-CREB signaling pathway-dependent manner (50). Interestingly, the role of *HCAR1* in brain cell proliferation was also reported in the repair process that follows neonatal brain injury. Lauritz et al. demonstrated, using the neurosphere assay, that brain cells lacking *HCAR1* had reduced proliferation and repair abilities (51). Moreover, *MCT1* is mainly used by oxidative cells for extracellular lactate intake, and *MCT4* is mainly used to release accumulated lactate into the extracellular milieu, in many cases by hypoxic and/or highly glycolytic cells (52–54). Our data supported the hypothesis that lactate leads GBM cells to increase the levels of *HCAR1* and *MCT1* in order to mediate lactate sensing (*HCAR1*) and lactate intake (*MCT1*) from the extracellular milieu. This phenomenon is coupled with increased mitochondrial content and fitness, thus prompting GBM cells toward oxidative metabolism. It is worth noting that this mechanism is not related to the increased lactate level itself, but is dependent on the interaction with the *HCAR1* receptor. Indeed, we were able to reproduce this metabolic reshaping using the selective *HCAR1* agonist 3,5-DHBA. Consistently, a study performed in *HCAR1*-silenced pancreatic cancer cells led to reduced mitochondrial activity and survival in several cancer cell (55). In particular, several cancer cell types, including colon, breast, lung, cervical, and pancreatic cancer cells, showed an increase in the expression of *HCAR1*, and functional studies indicated that this increase is important for lactate regulation of the genes involved in lactate uptake and metabolism. Moreover, *HCAR1* is critical for cancer cell survival only when glucose is absent and in the presence of lactate (56).

Interestingly, we observed major differences in the cell response to *HCAR1* activation when analyzing the levels of *MCT4*. Indeed, we observed that 24 h of lactate exposure mediated a reduction in the protein levels of *MCT4* in U-87 MG, whether we found a significant *MCT4* increase in both U-251 MG and A-172. It is noteworthy that the quantification of the main mitochondrial genes revealed that U-87 MG cells underwent a rapid increase of mitochondrial content, although less pronounced *versus* the basal levels, compared to that of A-172 and U-251 MG. Our data suggest that U-87 MG cells have different responses compared to the other cell lines in terms of timing to repurpose their transporters and metabolism. Indeed, upon 3,5-DHBA stimulation of *HCAR1*, we observed a significant increase of *MCT4* in U-87 MG, whereas we obtained contrasting results for A-172 and U-251 MG. Such a differential response of U-87 MG, characterized by a concomitant increase of *MCT1* and *MCT4*, may represent the molecular substrate leading to the heterogeneous response to *HCAR1* targeting in terms of E-cadherin activation among the tested cells. Further studies addressing the role of *MCT4* in epithelial–mesenchymal transition (EMT) and heterogeneity in the time–response to *HCAR1* activation between cell lines are needed.

This set of experiments suggests that the activation *HCAR1* induced the increase in *MCT1*, thus mediating lactate intake in stimulated cells. It is therefore conceivable that intercellular metabolism and mitochondrial content are closely related to the activation of *HCAR1* by several pathways. In this regard, Zaho et al. showed that increasing the lactate concentration in the liver TME could activate the *HCAR1* receptor and facilitate the *MCT1*-mediated uptake of lactate, leading to increased ATP production and decreased AMP/ATP ratio in the intracellular compartment (57). Tumor cells stimulate mitochondrial biogenesis not only for proliferation but also for promoting malignant transformation in the migration and invasiveness and during tumor adaptation to hypoxia (58, 59). As previously mentioned, we observed an increase of mitochondrial biogenesis in GBM cells treated with lactate or the *HCAR1* inducer. This phenomenon could be due to the increase of lactate uptake after *MCT1* overproduction. Moreover, we also showed that the increase of mitochondrial mass also induced an increase in *OXPHOS* gene expression. Exogenous treatment of lactate in various tumor cell lines induced an increase in ROS levels. We hypothesized that this latter increase in oxidative state enhanced mitochondrial biogenesis, similar to the increase in the expressions of *PGC1a* and *SIRT1* and oxidative genes.

Interestingly, our results also indicate that the activation of *HCAR1* promoted the modulation of β-catenin and E-cadherin expressions, suggesting that lactate participates in EMT in GBM. Several studies have been conducted to investigate the metabolic changes during EMT in breast, lung, and ovarian cancers, following an increased recognition of metabolic reprogramming as a hallmark of tumor development (60–62).

The lactate produced and exported by tumor cells can also be used by adjacent tumor cells in the TME, including endothelial cells and stromal cancer-associated fibroblasts, reprogramming their functions and contributing to tumor progression (63). Consequently, several authors hypothesized that lactate might also modulate the same epigenetic mechanisms in adjacent normal cells, including EMT processes (64, 65).

Given the insights from *in vitro* experiments on relevant human GBM cell lines, we employed a HRAS-overexpressing zebrafish model of GBM to examine whether similar metabolic changes are taking place in this model. Our data confirmed a widespread upregulation of glycolytic enzymes with the upregulation of *HCAR1*, thus indicating a prominent role in lactate signaling. In tumor cells, blockage of the MPC induced a reduction of the oxygen consumption in stress conditions and a massive reduction of the energy production through oxidative phosphorylation when lactate transport is inhibited, suggesting that lactate is an important source of energy in these cells. The increased expressions of the lactate transporter (*mct1*) and sensor (*hcar1*) were already present at 5 dpf, when tumors started to grow. Lactate exposure determined a significant increase in proliferating PH3-positive cells in RAS-overexpressing zebrafish brains, but not in control brains, and this was reverted by the selective inhibition of *MCT1*. This evidence suggests that lactate intake supports cell proliferation in cancer and that metabolic reshaping is a critical stimulus in the GBM microenvironment.

Thus, both cell culture and *in vivo* studies, using different approaches and different genes, converged toward the same conclusion, i.e., that glycolysis is prominent in GBM and leads to a massive production of lactate, which shapes the microenvironment toward an aggressive phenotype, providing energy substrates and representing a valid therapeutic target.

Our data from human GBM biopsies were also consistent with the preclinical evidence provided herein. We observed that, in high proliferative GBM biopsies, Ki67-negative cells expressed significantly higher levels of *MCT1* compared to proliferative cells and low proliferative GBM cells. This indicates that the response of GBM cells to lactate, besides sustaining metabolic reshaping and response, favored the proliferation of neighboring cells by cooperating with their glycolytic metabolism, sensing and removing extracellular lactate. Our data are consistent with those of other studies in patients with advanced cancer showing that *MCT1* inhibition may have a significant effect on cancer growth and progression and may represent a druggable target for the development of new therapeutic strategies [ClinicalTrials.gov identifier (NCT number): NCT01791595].

Further confirmation of our study results was obtained by analyzing the human GSE108474 dataset. The analysis allowed us to highlight that *MCT1* is significantly modulated during the progression of the disease. In particular, significant expression changes were highlighted with the increase in the degree of malignancy. Furthermore, our results showed that the expression of *MCT1* can potentially be used in order to discriminate patients with GBM *versus* those with astrocytoma and oligodendrocytoma. These data are in agreement with the current research study, which considers *MCT1* a new prognostic biomarker and a potential target in human GBM (66). Interestingly, the correlation analysis between the expression levels of *MCT1* and *IDH1* in GBM patients was inversely proportional, further confirming recently obtained data in which mutant *IDH1* expression was associated with the downregulation of monocarboxylate transporters (67).

## Conclusions

In conclusion, we showed that lactate is involved in various mechanisms favoring tumor development and progression. In particular, lactate has a dual role, being involved in the metabolic changes of tumor cells and acting as a molecule promoting cellular signaling through its membrane receptors. The ability of GBM cells to metabolically shift from glycolytic to oxidative metabolism, and *vice versa*, is likely to confer an advantage in survival, progression, and drug resistance. Firstly, glycolytic metabolism (the Warburg effect) supports fast cancer growth and provides an advantage for tumor proliferation. The lactate thus produced accumulates in the TME, which, on the one hand, favors the immune escape mechanisms and, on the other hand, modifies the metabolism of the adjacent tumor cells, also making them more resistant to antiblastic therapies. Therefore, lactate metabolism may be considered as a therapeutic target to develop novel pharmacological strategies for GBM therapy and to improve the outcome and quality of life of GBM patients.

## Data Availability Statement

The raw data supporting the conclusions of this article will be made available by the authors, without undue reservation.

## Ethics Statement

The studies involving human participants were reviewed and approved by the University of Catania. The patients/participants provided written informed consent to participate in this study. The animal study was reviewed and approved by the University of Trento.

## Author Contributions

NV, DT, GVB, AG, MM, and GV: conceptualization. LL, NV, DT, CG, AG, MM, and GV: project administration. LL, NV, DT, GB, MB, MR, RP, MM, and GV: methodology. LL, NV, DT, CG, GB, MB, and MR: investigation. LL, NV, DT, RC, GVB, MR, RP, AG, MM, and GV: formal analysis. DT, RC, RP, MM, and GV: resources. LL, NV, DT, RP, AG, MM, and GV: supervision. LL, NV, DT, MM, and GV: writing—original draft. LL, NV, DT, CG, GVB, MR, RP, AG, MM, and GV: writing–reviewing and editing. All authors contributed to the article and approved the submitted version.

## Funding

LL was supported by the International PhD Program in Neuroscience (Department of Biomedical and Biotechnological Sciences, University of Catania, Italy). This study was supported by Piano di Incentivi per la ricerca di Ateneo 2020/2022 Linea di intervento 2 (to GV). NV was supported by the PON AIM R&I 2014-2020-E66C18001240007, CG was supported by the PON AIM R&I 2014–2020-E68D19001340001, and MM was supported by BF 2020, provided by the CIBIO Department (University of Trento).

## Conflict of Interest

The authors declare that the research was conducted in the absence of any commercial or financial relationships that could be construed as a potential conflict of interest.

The reviewer FM declared a past collaboration/co-authorship with the author AG to the handling editor.

## Publisher’s Note

All claims expressed in this article are solely those of the authors and do not necessarily represent those of their affiliated organizations, or those of the publisher, the editors and the reviewers. Any product that may be evaluated in this article, or claim that may be made by its manufacturer, is not guaranteed or endorsed by the publisher.
