# Supplementary material for: Lactate Induces the Expressions of MCT1 and HCAR1 to Promote Tumor Growth and Progression in Glioblastoma
Source: Front Oncol. 2022 Apr 28;12:871798. doi: 10.3389/fonc.2022.871798 (PMC9097945; doi:10.3389/fonc.2022.871798)
Supplement: Supplementary file 1 [file DataSheet_1.docx]

Supplementary Material

## Supplementary Figures


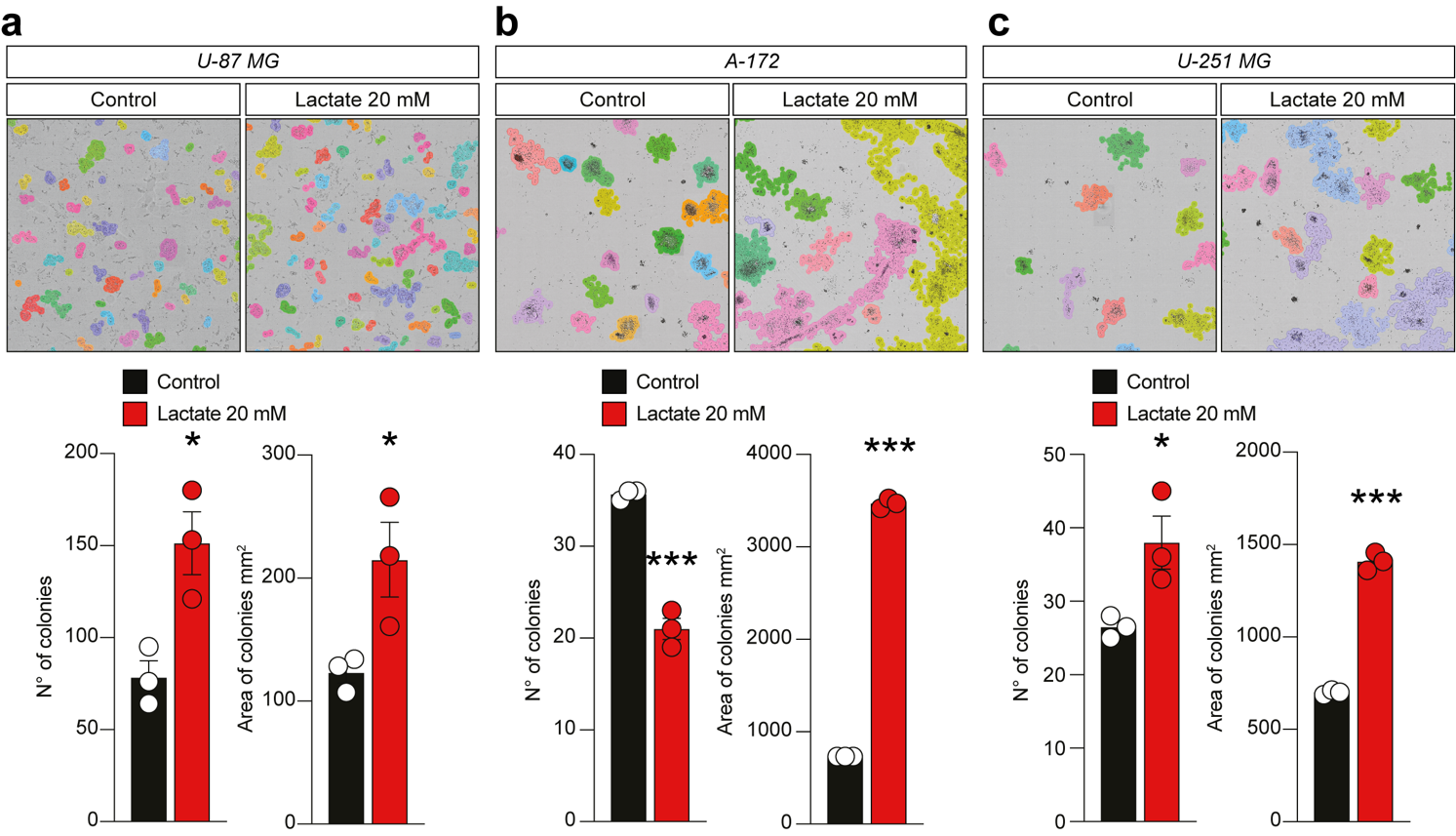


**Supplementary Figure 1. Lactate enhances colony formation capacity in glioblastoma cells.** Effect of Lactate treatment on colony formation capacity in U-87 MG cells (a), A-172 cells (b) and U-251 MG cells (c). Data are expressed as mean ± SEM of at least three independent experiments. (*p<0.05; **p<0.005; ***p<0.001; ****p<0.0001).


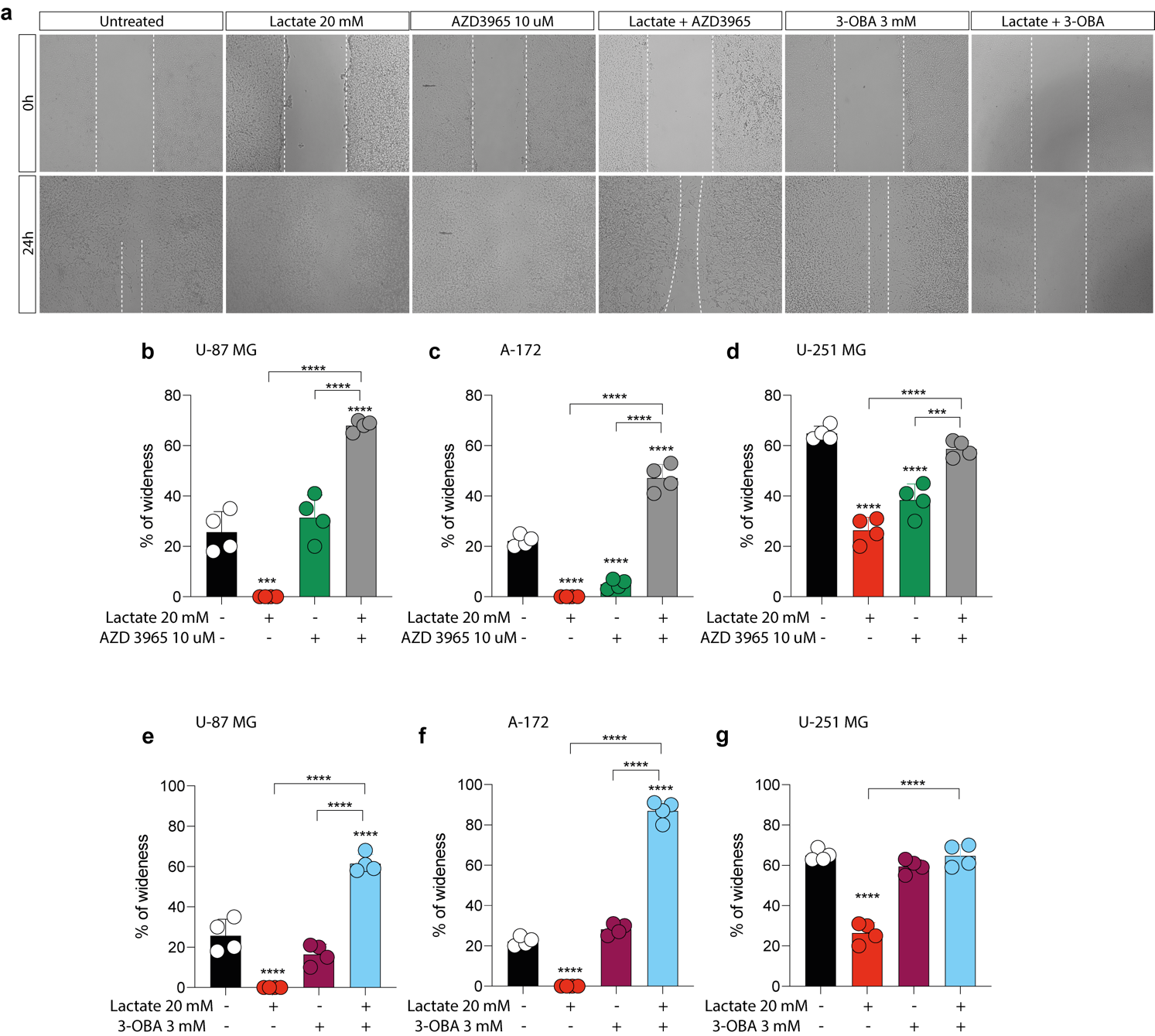


**Supplementary Figure 2. MCT1 inhibition and HCAR1 antagonism reduced migratory capacity in glioblastoma cells.** Effect of Lactate, AZD3965 and 3-OBA treatment on cell migration in U-87 MG cells (b,e), A-172 cells (a,c,f) and U-251 MG cells (d,g). Data are expressed as mean ± SEM of at least tfour independent experiments. (*p<0.05; **p<0.005; ***p<0.001; ****p<0.0001).


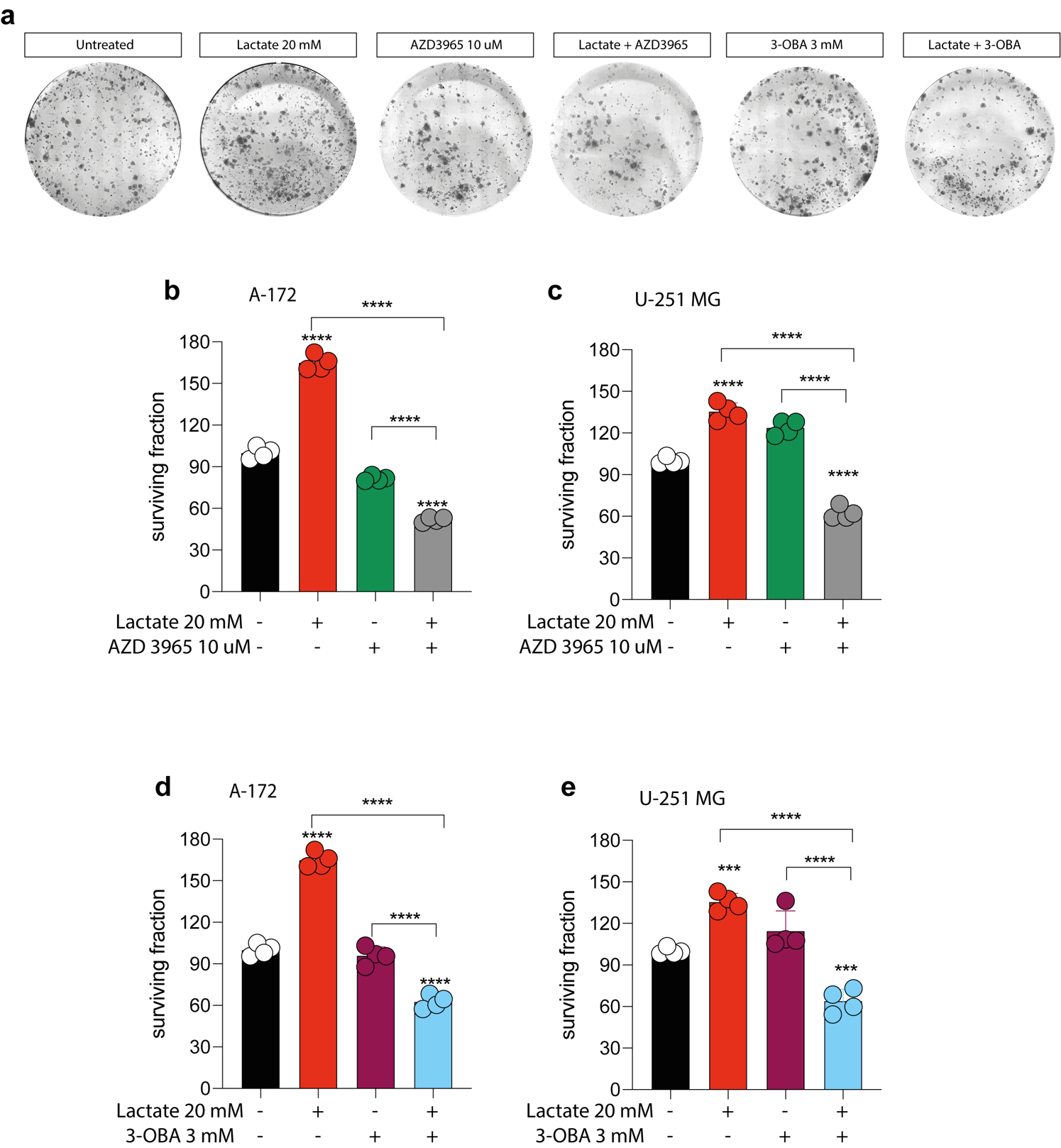


**Supplementary Figure 3. MCT1 inhibition and HCAR1 antagonism reduced the surviving fraction in glioblastoma cells.** Effect of Lactate, AZD3965 and 3-OBA treatment on surviving fraction in A-172 cells (a,b,d), and U-251 MG cells (c,e). Data are expressed as mean ± SEM of at least four independent experiments. (*p<0.05; **p<0.005; ***p<0.001; ****p<0.0001).
